# 3D contrast enhanced self navigated inversion recovery gradient echo coronary imaging in pediatric patients

**DOI:** 10.1186/1532-429X-17-S1-Q98

**Published:** 2015-02-03

**Authors:** Tim Slesnick, Gary R McNeal, Animesh Tandon, Denver Sallee, James W Parks, Michael O Zenge, Davide Piccini

**Affiliations:** 1 Department of Pediatrics, Emory University, Atlanta, GA, USA; 2 Children's Healthcare of Atlanta, Atlanta, GA, USA; 3 Customer Solutions Group, Siemens Medical Solutions USA, Inc, Malvern, PA, USA; 4 MR Product Innovation and Definition, Healthcare Sector, Siemens AG, Erlangen, Germany; 5 Advanced Clinical Imaging Technology, Siemens Healthcare IM BM PI, Lausanne, Switzerland; 6 Department of Radiology, University Hospital (CHUV), University of Lausanne (UNIL) and Center for BioMedical Imaging (CIBM), Lausanne, Switzerland

## Background

Coronary arterial imaging in pediatric patients is challenging for multiple reasons, including higher heart rates and smaller vessel sizes. 3D respiratory navigator inversion recovery gradient echo (NAV IR GRE) imaging after administration of a blood pool contrast agent has shown advantages compared to 3D T2 prepared bSSFP imaging. Both techniques, however, provide unreliable acquisition timing since respiratory navigation efficiency varies based on patient conditions. Self-navigated (SN) whole heart imaging using a T2 prepared bSSFP readout, with tracking of the intracardiac blood pool rather than the diaphragm resulting in 100% efficient data acquisition, has been previously introduced. This technique, however, has not been applied using an IR preparation and GRE readout (SN IR GRE). We therefore sought to apply the SN IR GRE methodology in pediatric patients.

## Methods

A previously described prototype 3D radial phyllotaxis SN pulse sequence was converted to perform an IR preparation pulse and GRE based readout. A series of iterative experiments was performed to optimize flip angle, bandwidth and inversion time after administration of gadofosveset trisodium. Once optimal parameters were determined, 10 patients underwent both respiratory navigated and self-navigated 3D imaging on a 1.5T scanner (Siemens MAGNETOM Aera). Images were assessed for coronary artery clarity, including quantitative assessment of SNR and CNR, vessel sharpness, and visualized length using the "Soap-Bubble" tool.

## Results

**Figure 1:**
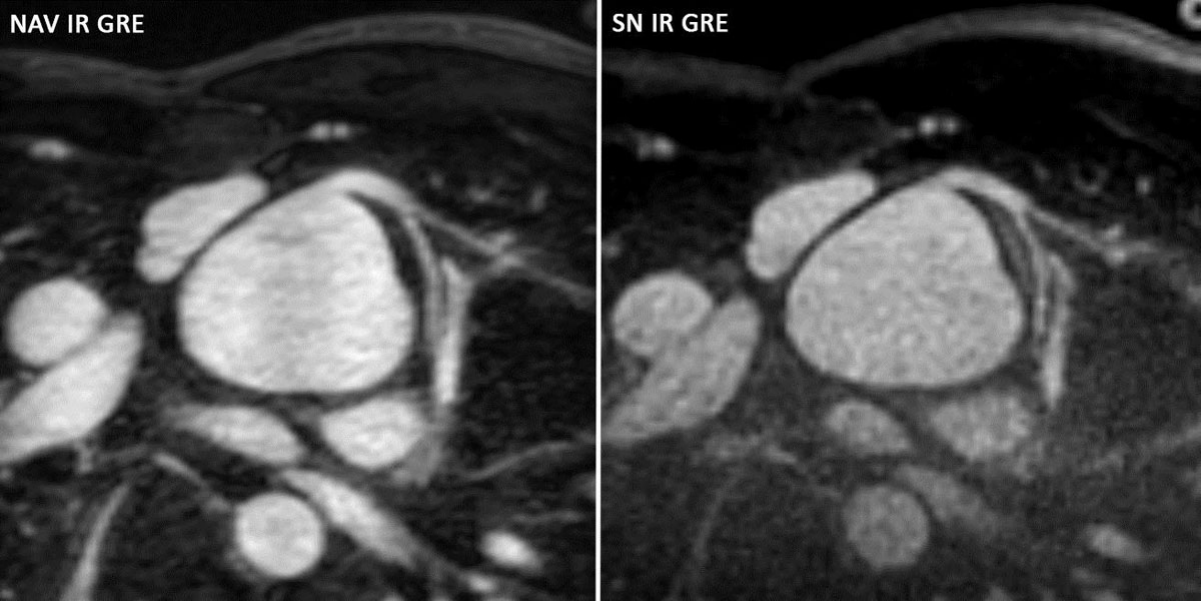
NAV IR GRE and SN IR GRE images of the LCA in a child with d-transposition of the great arteries who has undergone an arterial switch operation.

SN IR GRE image quality was optimized with flip angle = 15°, bandwidth = 1000 Hz/Px, and inversion time = 290 ms. The median age of the patient cohort was 15.1 years (range 7.1 - 17.7), with an average heart rate of 77±15 bpm. Diagnoses included Tetralogy of Fallot (5), coronary artery anomaly (2), bicuspid aortic valve (2), and d-transposition of the great arteries (1). Acquisition duration for the SN IR GRE was extremely predictable, with an inverse linear relationship with heart rate. Image quality was excellent for all patients with both methodologies (Figure 1), with diagnostic visualization of the coronary origins and proximal courses. On side-by-side qualitative comparison, the SN IR GRE was superior in 4, inferior in 4, and equivalent in 2 patients. Quantitative assessment of vessel sharpness and distance visualized are shown in Table 1. SNR and CNR were higher using the NAV IR GRE (p<0.01), but vessel sharpness and visualized length were not statistically different for the LCA and RCA.

**Table 1:** Quantitative analysis or NAV IR GRE vs SN IR GRE

Sequence	SNR	CNR	Visible RCA Length (mm)	Sharpness RCA	Visible LCA Length (mm)	Sharpness LCA
NAV IR GRE	169 ± 76	122 ± 54	59 ± 33	41 ± 8	68 ± 28	37 ± 15

SN IR GRE	75 ± 19	45 ± 11	60 ± 35	31 ± 15	79 ± 24	35 ± 13

p-value	0.004	0.002	0.96	0.09	0.35	0.77

All values given as mean +/- standard deviation.

## Conclusions

Self-Navigated IR GRE acquisitions are feasible in pediatric patients, with predictable acquisition times. Diagnostic quality is excellent, and though SNR and CNR are higher with NAV IR GRE, vessel sharpness and visualized length were not different for the coronary arteries.

## Funding

None.

